# Diabetic Nephropathy and COVID-19: The Potential Role of Immune Actors

**DOI:** 10.3390/ijms22157762

**Published:** 2021-07-21

**Authors:** Diane Mourad, Nadim S. Azar, Sami T. Azar

**Affiliations:** 1 Department of Internal Medicine, Endocrinology Division, Faculty of Medicine and Medical Center, American University of Beirut, Beirut 11-0236, Lebanon; diane.mourad@live.com; 2 Department of Anatomy, Cell Biology, and Physiological Sciences, Faculty of Medicine and Medical Center, American University of Beirut, Beirut 11-0236, Lebanon; nadimazar1996@gmail.com; 3 Endocrinology, Diabetes and Metabolism Division, American University of Beirut Medical Center, Beirut 11-0236, Lebanon

**Keywords:** diabetic nephropathy, COVID-19, immune actors, Neuropilin-1, ACE-2, mitochondrial glutathione, vitamin D, DPP4

## Abstract

Nowadays, type II diabetes mellitus, more specifically ensuing diabetic nephropathy, and severe COVID-19 disease are known to be closely associated. The exact mechanisms behind this association are less known. An implication for the angiotensin-converting enzyme 2 remains controversial. Some researchers have started looking into other potential actors, such as neuropilin-1, mitochondrial glutathione, vitamin D, and DPP4. In particular, neuropilin-1 seems to play an important role in the underlying mechanism linking COVID-19 and diabetic nephropathy. We suggest, based on the findings in this review, that its up-regulation in the diabetic kidney facilitates viral entry in this tissue, and that the engagement of both processes leads to a depletion of neuropilin-1, which was demonstrated to be strongly associated with the pathogenesis of DN. More studies are needed to confirm this hypothesis, and research should be directed towards elucidating the potential roles of all these suggested actors and eventually discovering new therapeutic strategies that could reduce the burden of COVID-19 in patients with diabetic nephropathy.

## 1. Introduction

It is recognized today that type II diabetes mellitus (T2DM) patients are at risk of a more aggressive course of the COVID-19 disease with a higher hospitalization rate and longer duration of hospital stay [1]. Indeed, several studies have already shown a close relationship between diabetes and mortality due to COVID-19, with 1.75 (pooled Odds Ratios (OR) = 1.75; 95% Confidence Interval (CI) 1.31–2.36; *p* = 0.0002) [2] to 2.52 (OR = 2.52; 95% CI = 1.93–3.30, *p* ≤ 0.00001) [3] times higher risk of death for diabetic patients versus the general population. COVID-19 disease severity is also significantly increased in patients with diabetes by almost the double (OR = 2.20; 95% CI = 1.69-2.86, *p* < 0.00001) [3].

Several pre-existing renal diseases are known risk factors for severe COVID-19 outcomes. For instance, in a very recent study, it was shown that severe Acute Kidney Injury (AKI) was present in 54% of COVID-19 deceased patients, and biopsy findings included Diabetic Nephropathy (DN) in 27% of the cases (DN referring to the deterioration of kidney function associated to diabetes, which can lead to chronic kidney disease and to mortality in diabetic patients) [4]. Moreover, end stage renal disease (ESRD) was found to be associated with a high mortality rate among hospitalized patients with COVID-19 [5].

Finally, patients with DN were shown to have nearly two-times higher rates of COVID-19 pneumonia and of intubation with higher probabilities of admission and death once admitted compared to patients with chronic kidney disease alone (with both diseases associated with higher rates of COVID-19 pneumonia, intubation and case-fatality versus the overall population) [6].

While there is supporting evidence of the association between DN and COVID-19 disease severity, there remains a scarcity of data on the underlying mechanisms behind this association. Indeed, we conducted a literature review to search for articles with “Diabetic Nephropathy” and “COVID19” (search term encompassing “Coronavirus disease 2019” and “SARS-CoV-2”) as keywords. A search of four relevant databases (PubMed, Scopus, Goggle Scholar and ClinicalTrials.gov) up to 24 May 2021 was then conducted for all relevant publications following the below search strategy:

- Search Terms: Diabetes Nephropathy AND COVID19 (1);
- Search Engines: Pubmed OR Scopus OR Google Scholar OR ClinicalTrials.gov (2);
- Search Combination: (1) AND (2);

This search yielded several results, and we will discuss in this narrative review the most interesting and relevant findings.

## 2. Discussion

### 2.1. The Well-Known ACE Role and Its Controversial Involvement

Early evidence has indicated that the primary host receptor of Severe Acute Respiratory Syndrome Coronavirus 2 (SARS-CoV-2, the etiologic agent of the COVID-19 pandemic) is the angiotensin-converting enzyme 2 (ACE2). This entry process is mediated by binding SARS-CoV-2 spike receptor-binding domain (RBD) domain to ACE2 [7].

Indeed, protruding from the viral surface is a densely glycosylated spike (S) protein, which mediates host cell entry by engaging ACE2 [8].

As already shown, a high number of hospitalized COVID-19 patients are diabetic and angiotensin-converting enzyme inhibitors (ACEIs) and angiotensin II receptor blockers (ARBs) are used, mainly for their nephroprotective effects, as first-line agents in diabetic patients. Nonetheless, their administration leads to up-regulation of ACE2, with a potential for increasing the viral entry of SARS-CoV-2 [9]. Furthermore, a reduction and/or inactivation of ACE2 caused by SARS-CoV-2 binding can increase the ACE/Angiotensin II signaling pathway and related pathologies [10]. Thus, the COVID-19 disease was shown to be characterized by ACE2 depletion, probably playing a key role in the devastating cytokine storm embodying this disorder [11].

Conversely, it was suggested that ACE2 has the potential of slowing the progression of experimental diabetic chronic kidney disease [11], and it was found to be highly and significantly expressed in the kidney among individuals with chronic kidney diseases or DN [12].

Indeed, diabetic patients with kidney disease revealed ACE2 expression in proximal tubular epithelial cells primarily [13], and ACE2 messenger RNA (mRNA) expression levels were significantly upregulated versus healthy living donors’ kidneys [14].

The increased ACE2 mRNA expression in the kidneys of diabetic patients may increase the severity and/or risk of kidney infection with SARS-CoV-2 in the setting of COVID-19 disease [15].

Several studies attempted to gain new insights into the pathogenic mechanisms of SARS-CoV-2 underlying this clinical manifestation in the kidney. Notably, receptors for proinflammatory cytokines, especially IL6ST, were found to be concentrated in renal endothelial cells, which suggests the occurrence of alternative damaging autoimmune mechanisms [12].

Other authors studying the molecular network modules induced in ACE2-expressing proximal tubular epithelial cells in DN found that they were associated with viral entry, immune activation, endomembrane reorganization, and RNA processing [13]. This cell module overlapped with expression patterns typically seen in SARS-CoV-2-infected cells, which suggests a possible interaction between the ACE2-coregulated proximal tubular epithelial cell expression program and SARS-CoV-2 infection processes [13]. Hence, studying the SARS-CoV-2 receptor networks can support risk stratification and therapeutic strategies for kidney damage related to COVID-19 [13].

Many outcomes indicate that triggering the ACE2/Angiotensin (1–7)/MasR axis may be nephroprotective in the context of AKI [11]. However, there are still conflicting outcomes that indicate it may accelerate renal damage in CKD and AKI under certain conditions [11].

It is noteworthy to mention that expression levels of ACE2 were found to be unaltered by exposures to renin-angiotensin-aldosterone system inhibitors (RAASi) in diabetic kidney disease [13].

Nonetheless, potential harm by RAASi may be caused by other mechanisms, such as the increase in the ACE2 receptor activity, the inefficiency of the counter regulatory axis in the lungs and the proinflammatory properties of ACE2-positive cells infected with SARS-CoV-2 [16]. Moreover, a recent study showed that captopril (an ACEI) had a significantly higher incidence of pulmonary adverse events compared with other ACEI as well as ARBs [9].

The use of RAASi should be done judiciously with careful consideration, until more definitive evidence becomes available. Additionally, specific medication’s adverse event profile, particularly captopril, should be taken into account [16].

### 2.2. The Less Well-Known NRP-1 Role and Its Remarkable Potential

As already discussed, the COVID-19 entry process is mediated by binding SARS-CoV-2 spike receptor-binding domain (RBD) domain to ACE2 [7] However, some studies have reported susceptibility to the virus in intra- and extra-pulmonary immune and non-immune cells lacking ACE2 [8]. This suggests that the S protein may exploit additional receptors for infection, such as the innate immune system, including C-lectin type receptors (CLR), toll-like receptors (TLR) and neuropilin-1 (NRP1), and the non-immune receptor glucose regulated protein 78 (GRP78) [8].

Recently, an increasing amount of research is directed to the newly identified receptor responsible for the SARS-CoV-2 entry: NRP1, with the interaction between the former’s spike RBD domain and the latter’s b1 domain [7].

NRP1 is a single-pass transmembrane receptor protein lacking enzymatic activity, with a large extracellular tail structured in several domains. This allows NRP1 to interact with multiple ligands with different signaling pathways through its co-receptors [17]. Indeed, it is a multifunctional transmembrane receptor for ligands that affects developmental axonal growth and angiogenesis, with implications in the nervous system’s development, immunity, cancer and several viral infections [18]. It has been suggested to be an immune checkpoint of T cell memory, and its immune function involvement is compelling, given the role of an exaggerated immune response in COVID-19 related disease severity and death [19].

**Figure 1:**
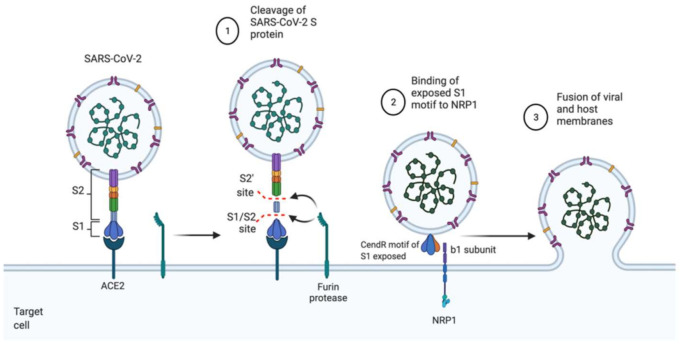
Mechanism of Cellular Entry of SARS-CoV-2 through NRP1. After binding of the spike protein to ACE-2 receptor, Furin-mediated cleavage of S protein at S1/S2 site leads to the exposure of the CendR motif of S1 which easily binds to the b1 subdomain of NRP1 receptor. This allows it to undergo membrane fusion and endocytosis. Abbreviations: ACE2, angiotensin-converting enzyme 2; CendR, C-end rule; NRP1, neuropilin 1.

As the Spike protein of SARS-CoV-2 is cleaved into the S1 and the S2 domain by furin protease, NRP1 binds, mainly through its b1 domain, to the newly created C-terminal amino acid sequence of the S1 domain [20]. The furin cleavage product of SARS-CoV-2 Spike protein takes advantage of the NRP1 vascular endothelial growth factor A (VEGF-A) binding site [18] (Figure 1).

Indeed, through its exposed C-end rule (CendR) motif following the furin processing, the SARS-CoV-2 spike protein binds to the NRP1 CendR pocket, which allows it to achieve endocytosis and cell entry. This binding interferes with that of NRP1’s endogenous ligand VEGF-A, a signaling that would otherwise promote nociception [21]. The ensuing silencing of pain through the VEGF-A pathway may underlie increased disease transmission in asymptomatic individuals [22].

Moreover, in the presence of NRP1 binding S1 more strongly to the host membrane, there is a high probability of S2 being pulled out. Hence, this NRP1 binding could stimulate the separation of S1 and S2 domains, which will probably increase the infectivity of SARS-CoV-2 as the liberated S2 mediates the fusion of the virus and other host membranes [23].

As such, NRP1 may increase viral infection by SARS-CoV-2 in the presence of other host factors like ACE2 [21], and it may also initiate receptor-dependent viral internalization, potentiate severe immune-pathological inflammation, and lead to a systemic spread of the infection [8], independently of ACE2. Nonetheless, recent in-vitro studies are showing that NRP1 alone did not increase cell susceptibility to viral infectivity [24], suggesting that it may enhance viral infectivity via other host factors, rather than mediate it itself. Further studies are required to clarify this mechanism.

Although originally found in neuronal cells, NRP1 is also expressed by other cells, namely in the kidney. It was indeed found that NRP1 is highly expressed in differentiated podocytes [25]. Some of NRP1’s ligands have been implicated in diabetes and in DN, but scarce data are available to date [17]. 

Prolonged and uncontrolled hyperglycemia leads to the accumulation of AGEs or Advanced Glycation End-products. These products play an important role in the pathogenesis of DN. Indeed, research showed that the addition of glycated Bovine Serum Albumin (AGE-BSA) to differentiated murine podocytes affected the migration ability of the podocytes by significantly reducing their adhesion to collagen IV, laminin, and fibronectin compared with non-glycated BSA-incubated cells [26]. The decreased migration ability may be linked to an increased adherence of some uncovered areas of the glomerular basement membrane to Bowman’s capsule which could lead to focal glomerulosclerosis [25].

It was suggested that the pathophysiology of AGE-challenged podocytes (hypertrophy, apoptosis, and reduced cell migration) is closely related to the inhibition of NRP1 [27]. Indeed, the addition of AGE-BSA to differentiated murine podocytes was shown to inhibit NRP1 expression by inhibiting NRP1 promoter transcriptional activity in podocytes via the reduction of the Sp1 transcription factor’s binding ability to attach to the NRP1 promoter [28].

In fact, it seems that, since the reduced podocyte migration related to the addition of AGE-BSA could be duplicated in the absence of AGE-BSA when NRP1 expression is down-regulated by short interference (si) RNA, AGE-BSA addition inhibits podocyte migration by down-regulating NRP1 [25]. Furthermore, podocyte migration could be stimulated by overexpressing NRP1, even in the presence of AGE-BSA, which re-emphasizes the hypothesis [25].

In a more recent study from the same authors, it was demonstrated that the NRP1 depletion reduced the phosphorylation of focal adhesion kinase (FAK) and of Erk1/2 in differentiated podocytes. It could also inhibit the activation of the Rac-1 and Cdc42 GTPase activity [26]. A role for NRP1 in the regulation of podocytes’ adhesion to extracellular matrix proteins, actin cytoskeleton reorganization, and apoptosis was also shown (17). These NRP1-induced effects may be responsible for the podocytes damage and loss in DN [26].

In this sense, several studies have proven a reduced expression of NRP1 in glycated-BSA cultured differentiated podocytes as well as in glomeruli from db/db mice (a model of T2DM) and in diabetic patients diagnosed with DN [17].

It is important to mention that in a recent analysis of an RNA sequencing dataset of cryopreserved human diabetic kidney, of the numerous proposed factors implicated in the cell entry of SARS-CoV-2, including ACE2, only NRP1 was significantly up-regulated [29]. Up-regulation of NRP1 in the diabetic kidney cells suggests its importance in a population at high risk of severe COVID-19 disease. It is also unknown whether the NRP1 up-regulation and involvement in COVID-19 may have direct implications for the disease’s outcomes and long-term consequences, including possible immune dysfunction [19].

Therefore, a logical hypothesis would be that the up-regulation of NRP1 in the diabetic kidney could facilitate the entry of SARS-CoV-2 in this tissue and that the engagement of the two could lead to depletion of NRP1 with progression to podocyte damage, and ultimately, to DN. More research is needed to refine the current understanding of the potential role of NRP1 in DN and COVID-19.

### 2.3. Other Suggested Underlying Actors

#### 2.3.1. Mitochondrial Glutathione

Mitochondria are known to be the main source of ROS or reactive oxygen species, mainly deriving from the mitochondrial respiratory chain. Among the several mitochondrial enzymatic and non-enzymatic antioxidant systems, mitochondrial glutathione (mGSH) appears as the key line of defense for preserving an appropriate mitochondrial redox environment. mGSH can act directly or as a co-factor in numerous reactions catalyzed by other mitochondrial enzymes making it vital to repair or even evade oxidative modifications which could impact mitochondrial function and even subsequently lead to cell death [30].

Since mitochondrial ROS can lead to deregulated inflammatory responses, including proinflammatory cytokine production [31], in conditions with excessive inflammatory response, as seen in severe COVID-19 symptoms, it was suggested that mitochondrial antioxidants, such as mGSH, could play a role during the COVID-19 viral infection [30].

In fact, the morbidity and mortality of SARS-CoV-2 are mainly due to severe cytokine storm and hypercoagulable state caused by the host’s dysregulated inflammatory immune response, which leads eventually to multi-organ failure. Therefore, it was suggested that people with depleted GSH levels are prone to mortality from COVID-19 [32] and that GSH supplementation should be used as add-on to the current treatment options in COVID-19 patients [33], as the mechanisms leading to deadly inflammation could be counterbalanced by GSH [34].

It was also shown that oxidative stress and nitrosative stress both play a major role in the mechanism by which chronic hyperglycemia causes cellular damage to the kidneys [35], and low levels of renal GSH have been associated with DN [36].

Maintaining optimal levels of mGSH is therefore vitally important, and in fact, it has been demonstrated that dietary GSH supplementation could protect partially against many of the DN-related pathological changes [37].

A possible role for mGSH involvement in both diseases could be strengthened and requires further research.

#### 2.3.2. Vitamin D

Vitamin D is a crucial hormone that regulates calcium and phosphate homeostasis affecting bone growth and turnover and has many other functions via its gene transcription effects, acting, among others, as a regulator of the immune system [38]. Vitamin D deficiency is a very common disorder and is linked to many diseases, including T2DM and DN but also more recently, COVID-19 disease [39].

A role for vitamin D in the response to COVID-19 infection could be speculated, first, via the production of antimicrobial peptides in the respiratory epithelium it supports, which could make the viral infection and development of symptoms less likely, and second, via the reduction of the inflammatory response and the dampening of the cytokine storm [38].

Therefore, vitamin D deficiency has been linked to a susceptibility to COVID-19 and to worse clinical outcomes, as a majority of hospitalized COVID-19 patients were diagnosed with vitamin D insufficiency [40].

Finally, vitamin D administration to deficient individuals was hypothesized to prevent COVID-19 infection and/or alter the course of disease severity. Immune dysregulation is a key feature of severe COVID-19. Indeed, vitamin D’s roles in initially controlling viral infection and later reducing the hyper-inflammation may allow the restoration of the immune balance to prevent the cytokine storm and to combat COVID-19 disease severity [41].

Several lines of evidence have indicated that vitamin D deficiency is associated with T2DM [42] but also that a tight relationship exists between vitamin D deficiency and DN, with vitamin D-deficient diabetic patients appearing to be at a higher risk of DN [43]. Multiple roles of vitamin D in podocyte injury, tubule lesions, interstitial fibrosis, inflammation, etc. have been demonstrated [44], and the robust anti-inflammatory properties of vitamin D render its supplementation a promising nephroprotective therapeutic option for DN [45].

In fact, it was shown that increased serum vitamin D levels reduce blood glucose levels and increase insulin secretion. Vitamin D may also help to prevent DN by reducing the production of GFAT or Glutamine: Fructose-6-phosphate Aminotransferase as the main enzyme of the hexosamine pathway in renal tissue [46].

Hence, vitamin D appears to be a second interesting underlying actor in both COVID-19 and DN and deserves more direct studies on both diseases.

#### 2.3.3. Dipeptidyl Peptidase-4

Dipeptidyl peptidase-4 (DPP4), also known as the T cell activation antigen CD26, is a serine membrane-anchored ectopeptidase expressed ubiquitously on the surface of different cell types including those present in the kidneys, the respiratory tract and the immune system. It has a catalytic activity as it cleaves dipeptides from the N-terminus and acts as a binding protein as well as a ligand of extracellular factors [47].

DPP-4 cleaves incretins including GLP-1 or glucagon-like peptide-1 and GIP or glucose-dependent insulinotropic polypeptide, which leads to reduced insulin secretion and abnormal visceral adipose tissue metabolism, but also regulates postprandial glucose. Its expression is higher in visceral adipose tissue and directly correlates with insulin resistance and adipocyte inflammation. Hence, DPP-4 inhibitors are widely used to treat T2DM [48].

Human DPP-4 has already been implicated in the Middle East respiratory syndrome coronavirus as a functional receptor for the spike glycoprotein. Indeed, post-translational N-terminal hypersialylation may play an important role in the DPP4 trafficking and virus aggressivity and could comprise the N-glycan binding interfaces of DPP4 [47].

Its ancestor, SARS-CoV-2, may be using the DPP4 receptor as co-receptor to the ACE2 [47] when entering the host’s cells. A large interface has been predicted in the docking of DPP-4/SARS-CoV-2 spike protein [49], and it seems that the S1 domain of COVID-19 spike glycoprotein may interact with the human DPP4, a key immunoregulatory factor for hijacking and virulence [50].

DPP-4 appears to accelerate SARS-CoV-2’s entry into the respiratory airways but also its spread to other tissues, such as the kidneys, and can contribute in the cytokine storm and immunopathogenesis [47]. Some authors hypothesize that DPP4 inhibitors could represent a new strategy to support the treatment of COVID-19 in patients, with or without diabetes, by reducing the viral entry and replication into the respiratory tract and by hampering the inflammation and cytokine storm within the lungs [47].

DPP-4 expression was found to be upregulated in the glomeruli of patients with diabetic kidney disease (DKD) [51]. The immune system’s involvement in its pathogenesis and the contribution of renal inflammation in advanced DKD are now well identified [52]. DPP-4 has been shown to increase inflammation in T2DM as its enzymatic activity affects the function of several cytokines, chemokines, and growth factors [53].

Beyond their effect on glycemic control, emerging evidence suggests that DPP-4 inhibitors may have additional effects such as nephroprotection [54].

For instance, a study examining the long-term nephroprotective effects of a DPP-4 inhibitor in db/db mice, a model of T2DM, showed it can delay the progression of DN damage in a glucose- and blood pressure-independent manner. The observed effects were attributed to the attenuation of podocyte injury and the inhibition of the transformation of myofibroblasts [51].

With this available evidence, the anti-inflammatory properties of DPP-4 inhibitors suggest their potential implication in DN and COVID-19 immunopathogenesis, and DPP-4 represents a potential target to reduce the pathological progression of both diseases [49].

## 3. Conclusions

Patients with diabetes are at high risk of severe COVID-19 disease progression, and patients with Diabetic Nephropathy are at an even higher risk. While this fact is well recognized today, the underlying mechanisms behind this have not yet been fully uncovered. Several actors are hypothesized to play a role, such as the ACE receptor and the RAAS axis, but also the immune system, mainly through NRP1. Based on the data available so far, we suggest that the up-regulation of NRP1 in the kidney of diabetic patients facilitates the entry of SARS-CoV-2 in this tissue, followed by an engagement of the two processes leading to a depletion of NRP1. Lower levels of NRP1 were demonstrated to be strongly associated with the pathogenesis of DN, further corroborating the association. Other potential actors may also contribute to the link between COVID-19 and DN, such as mitochondrial glutathione, vitamin D, and DPP4. Further research is needed in order to better understand the potential roles of all these suggested actors, especially NRP1 in DN and COVID-19. This could eventually lead to new therapeutic strategies that could lessen the burden of COVID-19 in patients with DN.

## Data Availability

No new data were created or analysed in this study. Data sharing is not applicable to this article.
